# 

**Published:** 1857-09

**Authors:** 


### CONTENTS.

ORIGINAL ESSAYS AND COMMUNICATIONS.

Filling Teeth. By J. Taft,.................................................. 5
Dental Chemistry of the Mouth. By Geo. Watt,.................................. 10
Richardson on Malposition,.................................................. 19
Paine on Treating Gold Fillings, &c.,.......................................... 20
Irregularity—Correction. By Dr. W. W. Allport,................................ 21
Partial Sets of Artificial Teeth. By H. R. Smith,.............................. 23

PROCEEDINGS OF SOCIETIES.

American Dental Convention,.................................................. 28

MISCELLANEOUS.

Filling Labial Surfaces of Upper Incisiors. By A. J. Volck, D. D. S.,............ 104
Biographical Sketch of Prof. Shotwell,.......................................... 106
Editorial.................................................................. 112

